# Use of an *In Vivo* FTA Assay to Assess the Magnitude, Functional Avidity and Epitope Variant Cross-Reactivity of T Cell Responses Following HIV-1 Recombinant Poxvirus Vaccination

**DOI:** 10.1371/journal.pone.0105366

**Published:** 2014-08-29

**Authors:** Danushka K. Wijesundara, Charani Ranasinghe, Ronald J. Jackson, Brett A. Lidbury, Christopher R. Parish, Benjamin J. C. Quah

**Affiliations:** 1 Molecular Mucosal Vaccine Immunology Group, The John Curtin School of Medical Research, The Australian National University, Canberra, Australia; 2 Cancer and Vascular Biology Group, Dept Immunology, The John Curtin School of Medical Research, The Australian National University, Canberra, Australia; 3 Alternatives to Animals through Bioinformatics Group, Dept Genome Biology, The John Curtin School of Medical Research, The Australian National University, Canberra, Australia; Instituto Butantan, Brazil

## Abstract

Qualitative characteristics of cytotoxic CD8^+^ T cells (CTLs) are important in measuring the effectiveness of CTLs in controlling HIV-1 infections. Indeed, in recent studies patients who are naturally resistant to HIV-1 infections have been shown to possess CTLs that are of high functional avidity and have a high capacity to recognize HIV epitope variants, when compared to HIV-1 infection progressors. When developing efficacious vaccines, assays that can effectively measure CTL quality specifically *in vivo* are becoming increasingly important. Here we report the use of a recently developed high-throughput multi-parameter technique, known as the fluorescent target array (FTA) assay, to simultaneously measure CTL killing magnitude, functional avidity and epitope variant cross-reactivity in real time *in vivo*. In the current study we have applied the FTA assay as a screening tool to assess a large cohort of over 20 different HIV-1 poxvirus vaccination strategies in mice. This screen revealed that heterologous poxvirus prime-boost vaccination regimes (i.e., recombinant fowlpox (FPV)-HIV prime followed by a recombinant vaccinia virus (VV)-HIV booster) were the most effective in generating high quality CTL responses *in vivo*. In conclusion, we have demonstrated how the FTA assay can be utilized as a cost effective screening tool (by reducing the required number of animals by >100 fold), to evaluate a large range of HIV-1 vaccination strategies in terms of CTL avidity and variant cross-reactivity in an *in vivo* setting.

## Introduction

Since the discovery of HIV as the causative agent for AIDS in 1983, the search for an effective vaccination strategy to control global HIV-1 progression has generated many promising yet disappointing outcomes [1]–[4]. Notable immune correlates in ‘elite controllers’ of HIV-1, however, may give clues as to what parameters should be targeted in rational vaccine design. In particular the presence of CTLs with high functional avidity and broad epitope variant cross-reactivity has been associated with HIV-1 control in elite controllers [5]–[7]. It is postulated that these CTL parameters could be a hallmark of effectively controlling HIV-1 infection.

Many of the current assays used to predict the efficacy of T cell immunity following vaccination provide limited insight with regards to functional avidity and epitope variant cross-reactivity. Moreover, currently available *in vivo*-based T cell function assays have limited capacity to effectively measure both functional avidity and epitope variant cross-reactivity [8]–[10]. Hence, we have recently developed a high-throughput multi-parameter *in vivo* T cell assay using the FTA technology that has the ability to measure functional avidity and epitope variant cross-reactivity with greater efficiency [11], [12]. This technique involves labeling of leukocyte target cells with carboxyfluorescein succinimidyl ester (CFSE), Cell Trace Violet (CTV) and Cell Proliferation Dye eFluor670 (CPD) to generate over 250 unique target cell clusters that can be injected into animals to monitor T cell responses in real time *in vivo*. This technique allows for the measurement of parameters such as cumulative magnitude of killing, functional avidity and epitope variant cross-reactivity of CTLs responding against FTA targets pulsed with peptides that bind to major histocompatibility complex (MHC) class I (MHC-I) molecules. In addition, the FTA assay also allows CD4^+^ T helper (T_H_) cell activity to be measured based on the ability of T_H_ cells to activate (e.g., up-regulate CD69 expression) FTA B cells pulsed with MHC class II (MHC-II) binding peptides.

In previous studies Ranasinghe *et al* have established that the combination of vaccine route and the order in which HIV-1 pox viral vaccine vectors are delivered in a prime-boost vaccination strategy, can alter magnitude and also the quality of HIV-specific CTL immunity [13]–[15]. In the current study the FTA assay was used to further clarify which pox-viral vector combination induced the best T cell immune outcome in terms of cumulative magnitude, functional avidity and epitope variant cross-reactivity of T cell responses *in vivo* following HIV-1 poxvirus prime-boost vaccination. The primary aim of the current study was to test the feasibility of using the FTA assay as a high-throughput vaccine screening tool to measure vaccine efficacy in animal models with the hope of establishing the best HIV-1 prime-boost vaccine vector combination that has potential to succeed into pre-clinical evaluation.

## Results

### Utility of the FTA assay in measuring T cell responses *in vivo*

**Figure 1:**
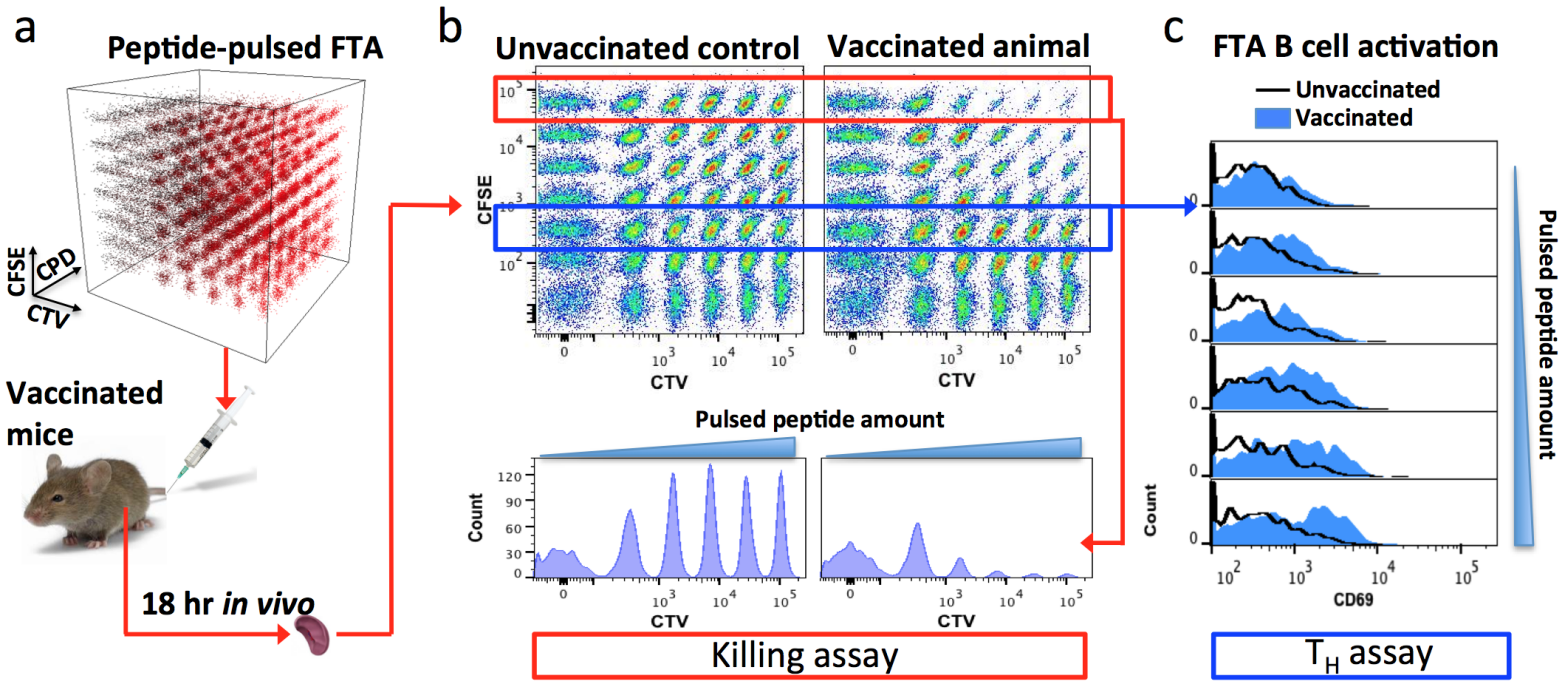
Schematic representation of a 252-parameter FTA assay. Splenocytes from mice were labeled with combinations of CTV (0 nM, 350 nM, 1295 nM, 4792 nM, 17729 nM and 65595 nM), CFSE (0 nM, 79 nM, 315 nM, 1106 nM 3859nm, 13505nm and 47269 nM) and CPD (0 nM, 106 nM, 690 nM, 2738 nM, 10262 nM and 38506 nM) to generate 252 discernable cell clusters. Cell clusters were pulsed with MHC-binding peptides (as outlined in Figure 3) to generate a panel of 42 peptide pulsed clusters and this repeated 6 times to generate 6 intra-animal repeats (i.e., 252 target cell clusters in total). Target cells were also labeled with DiI for discrimination from host splenocytes (not shown). (a) FTA cells were injected i.v. into host mice that had 6 days earlier been vaccinated with VV-HIV or left unvaccinated as a control. 18 hr after FTA injection, splenocytes were collected and target cells delineated from host splenocytes by DiI label using flow cytometry (not shown). (b) 2D plots of the fluorescence intensities of a panel of one replicate of the 42 peptide-pulsed clusters from the unvaccinated and vaccinated animals and an associated histogram analysis of clusters pulsed with titrations of the F2L CTL epitope, revealing killing in vaccinated animals. (c) An example of histogram analysis of the FTA T helper assay, with B220^+^ FTA cells pulsed with the Gag Th T_H_ cell epitope being assessed for CD69 up-regulation from the vaccinated animal compared to those from the unvaccinated animal.

**Figure 2:**
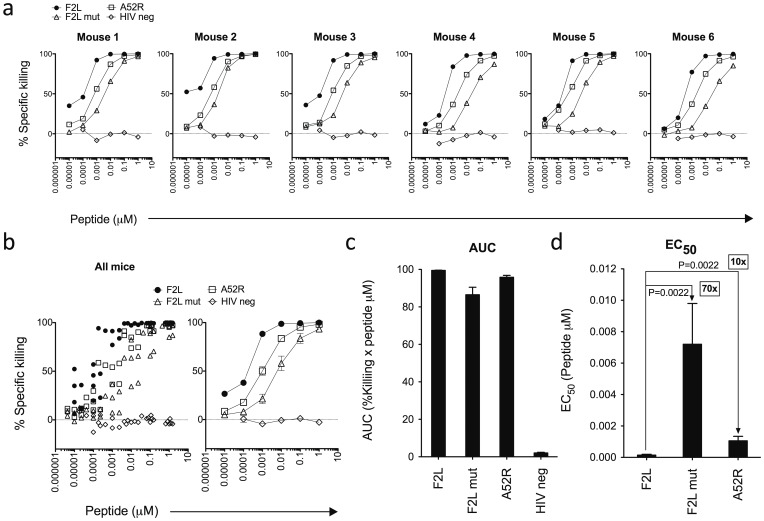
The FTA assay can measure magnitude, functional avidity and epitope variant cross-reactivity of CTL responses *in vivo*. Six BALB/c mice were vaccinated with 5×10^6^ PFU VV Western Reserve i.p. A FTA was constructed using mouse splenocytes and comprised of fluorescent target cells pulsed with 6 different concentrations of the MHC-I binding peptides F2L, F2L mut, A52R, and HIV neg (as a negative control). FTA target cells were injected i.v. into infected mice 6 days post vaccination and after 18 hr *in vivo* % specific killing calculated for FTA target cells from harvested spleens. a) *In vivo* killing responses from six infected animals. b) Summary of responses from all mice with means of % specific killing and standard error of mean. c) Mean area under curve (AUC) measurements from % specific killing response curves and associated standard error of means. d) Mean effective concentration of peptides used to pulse target cells that generated half maximal responses (EC_50_) and associated standard error of means and P values.

To establish the utility of the FTA assay, six BALB/c mice were immunized with wild-type vaccinia virus (VV) and *in vivo* killing responses to VV epitopes were assessed using the assay. Responses were assessed against the VV CTL epitopes F2L, the VV dominant epitope, F2L mut, a variant of F2L derived from modified vaccinia Ankara virus, and A52R, a subdominant VV epitope. A schematic representation of the steps involved in this technique is depicted in **Figure 1**. As anticipated, greatest CTL killing was observed against targets pulsed with immunodominant F2L, followed by responses to the subdominant epitope A52R (**Fig. 2a and 2b**). Despite not being present in the VV used for infection, the F2L mut epitope was also recognized by CTLs in infected animals demonstrating an epitope variant cross-reactive response (**Fig. 2a** **and** **Fig. 2b**). CTL responses were not detected against targets pulsed with the control HIV neg epitope, which is also not expressed by wild-type VV (**Fig. 2a and 2b**). These trends can be summarized by measuring the area under each curve (AUC) of the killing response, an indicator of the overall magnitude of CTL killing (**Fig. 2c**). In addition, the functional avidity of the CTL killing response was determined by calculating the effective concentration of peptide used to pulse the target cells required to generate half maximal killing (EC_50_) (**Fig. 2d**). This revealed that CTLs required 10 times the amount of A52R peptide to generate half maximal killing compared to F2L (**Fig. 2d**). Epitope variant cross-reacting CTLs required 70 fold higher amounts of F2L mut peptide than F2L to generate half maximal killing (**Fig. 2d**). These data provide an example of the utility of the FTA assay in measuring effector T cell response magnitude, functional avidity and epitope variant cross-reactivity in individual animals and highlights its reproducibility.

### Screening of VV- and FPV–based prime-boost vaccine regimes using the FTA assay

Having established the utility of the FTA assay, we applied the technique to screen for various HIV-1 poxvirus prime-boost vaccination regimes for their ability to induce CTL and T_H_ cell responses. To assess this we generated a vaccine regime matrix based on all the combinations (i.e., 24 combinations) of two vectors fowl pox virus (FPV)-HIV and VV-HIV given either i.n. or i.m. as a single vaccination or prime-boost strategy (**Table 1**). Immune responses to 7 different T cell epitopes were investigated, including two vaccinia CTL epitopes (F2L, F2L mut) as positive controls for robust CTL responses and four HIV CTL epitopes (HIV Gag, HIV Pol, HIV Env and HIV Gag mut, a HIV Gag subtype C variant of HIV Gag [25] not present in the vaccines and included to assess for epitope variant cross-reactive responses). Also a HIV Gag MHC-II binding epitope known as Gag T_H_, which we have previously shown to allow activation of B cell targets, was also included to measure T_H_ cell responses [12].

**Table 1:** HIV-1 pox virus vaccination regimes used in this study.

	PRIME VACCINATION				
BOOST VACCINATION	1 Nil	2 i.n. FPV	3 i.m. FPV	4 i.n. VV	5 i.m. VV
	6 i.n. FPV	7 i.n. FPV	8 i.m. FPV	9 i.n. VV	10 i.m. VV
		i.n. FPV	i.n. FPV	i.n. FPV	i.n. FPV
	11 i.m. FPV	12 i.n. FPV	13 i.m. FPV	14 i.n. VV	15 i.m. VV
		i.m. FPV	i.m. FPV	i.m. FPV	i.m. FPV
	16 i.n. VV	17 i.n. FPV	18 i.m. FPV	19 i.n. VV	20 i.m. VV
		i.n. VV	i.n. VV	i.n. VV	i.n. VV
	21 i.m. VV	22 i.n. FPV	23 i.m. FPV	24 i.n. VV	25 i.m. VV
		i.m. VV	i.m. VV	i.m. VV	i.m. VV

24 different vaccination regimes were included based on all combinations of FPV-HIV and VV-HIV administered either i.n. or i.m. in a prime-boost format. A control naïve animal receiving no vaccination (Nil) was also included.

**Figure 3:**
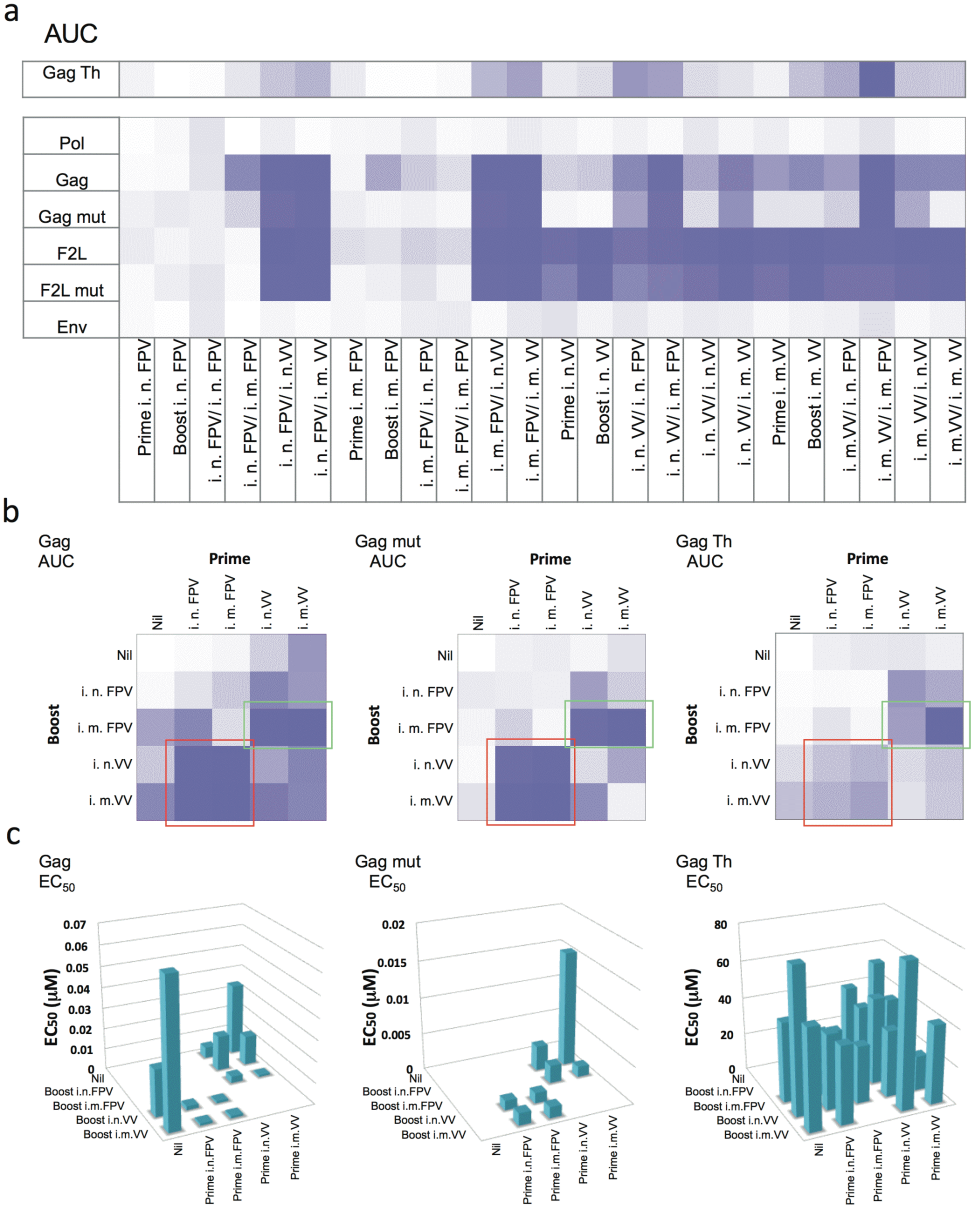
High throughput screening of HIV-1 poxvirus vaccination regimes for high magnitude, high functional avidity and high epitope variant cross-reactive T cell responses *in vivo*. Mice were vaccinated with 24 different vaccine regimes based on all combinations of FPV-HIV and VV-HIV administered either i.n. or i.m. in a prime-boost strategy as outlined in Table 1. Mice were vaccinated with 5×10^6^ PFU of each vaccine and booster vaccinations were given 2 weeks after the priming vaccination. T cell responses were assessed using a 252-parameter FTA comprised of fluorescent target cells pulsed with 6 concentrations of the MHC-I binding peptides F2L, F2L mut, HIV Gag, HIV Gag mut, HIV Pol and HIV Env, and the MHC-II binding peptide Gag T_H_. FTA target cells were injected i.v. into vaccinated mice 6 days post vaccination and responses assessed after 18 hr *in vivo* using harvested spleens. % specific killing and T_H_ cell activity was calculated as described in the Materials and Methods. a) Cumulative magnitude of T cell responses as AUC was plotted as a heat map depicting the range of highest (darkest colour) to lowest (lightest colour) T_H_ cell (upper panel) or CTL (lower panels) responses. b) AUC heat maps of responses to the three HIV Gag epitopes. c) EC_50_ values of responses to the three HIV Gag epitopes. Values are only shown where EC_50_ values were calculable as described in the Materials and Methods. AUC and EC_50_ values are depicted as means from 6 intra-animal replicates. The results are representative of three independent experiments

Splenocytes from mice vaccinated using the various regimes described in **Table 1** were assessed for FTA cell death and B cell activation to measure CTL activity and T_H_ responses respectively (See Figure 1c for T_H_ response assessment). A representative result from three independent experiments is shown in **Figure S1a, b, c, d, and e** and includes data from six intra-animal replicates totaling 6048 data points per experiment. Due to the large amount of data generated in this instance, AUC values were calculated for responses against each epitope and this depicted as a heat map to allow trends to be clearly revealed (**Fig. 3a**). From the heat map it was clear that several vaccine regimes gave killing responses to the HIV Gag epitope and even the HIV Gag mut epitope, approaching those generated to F2L mut in VV vaccine regimes. In contrast, negligible killing responses to HIV Pol and HIV Env epitopes were present. Furthermore, there were also several vaccine strategies that gave robust T_H_ cell responses to the Gag T_H_ epitope. Focusing on the three HIV Gag epitopes revealed two notable clusters of vaccine regimes giving high magnitude T cell responses, which corresponded to heterologous poxvirus prime-boost vaccine strategies (**Fig. 3b** **highlighted in red and green**). Of particular note was a cluster of four heterologous prime-boost vaccine regimes comprised of FPV-HIV prime and followed by VV-HIV booster vaccination (**Fig. 3b** **highlighted in red**). These vaccine strategies elicited high magnitude killing of targets presenting the HIV Gag epitope, and similarly the HIV Gag mut epitope. These responses were indicative of robust Gag epitope variant cross-reactive CTL responses following vaccination.

The functional avidity analysis of the responses to the two CTL HIV Gag epitopes revealed that the FPV-HIV/VV-HIV prime-boost immunization cluster also generated CTL responses of greatest avidity as these regimes showed the lowest EC_50_ killing responses compared to other vaccination regimes tested (**Fig. 3c**). No route dependent differences were detected in this cluster. The second notable cluster in terms of generating high quality CTL responses was that of two heterologous vaccine strategies comprised of VV-HIV prime followed by i.m. FPV-HIV booster vaccination (**Fig. 3b highlighted in green and 3c**). While these two clusters of heterologous prime-boost vaccination regimes also generated T_H_ cell responses to the Gag T_H_ epitope, negligible avidity changes were apparent with these MHC-II-restricted T_H_ cell responses (**Fig.3b and 3c**). Thus, overall the best vector combination in terms of inducing high quality CTL cell responses was found to be heterologous prime-boost vaccination regimes.

**Figure 4:**
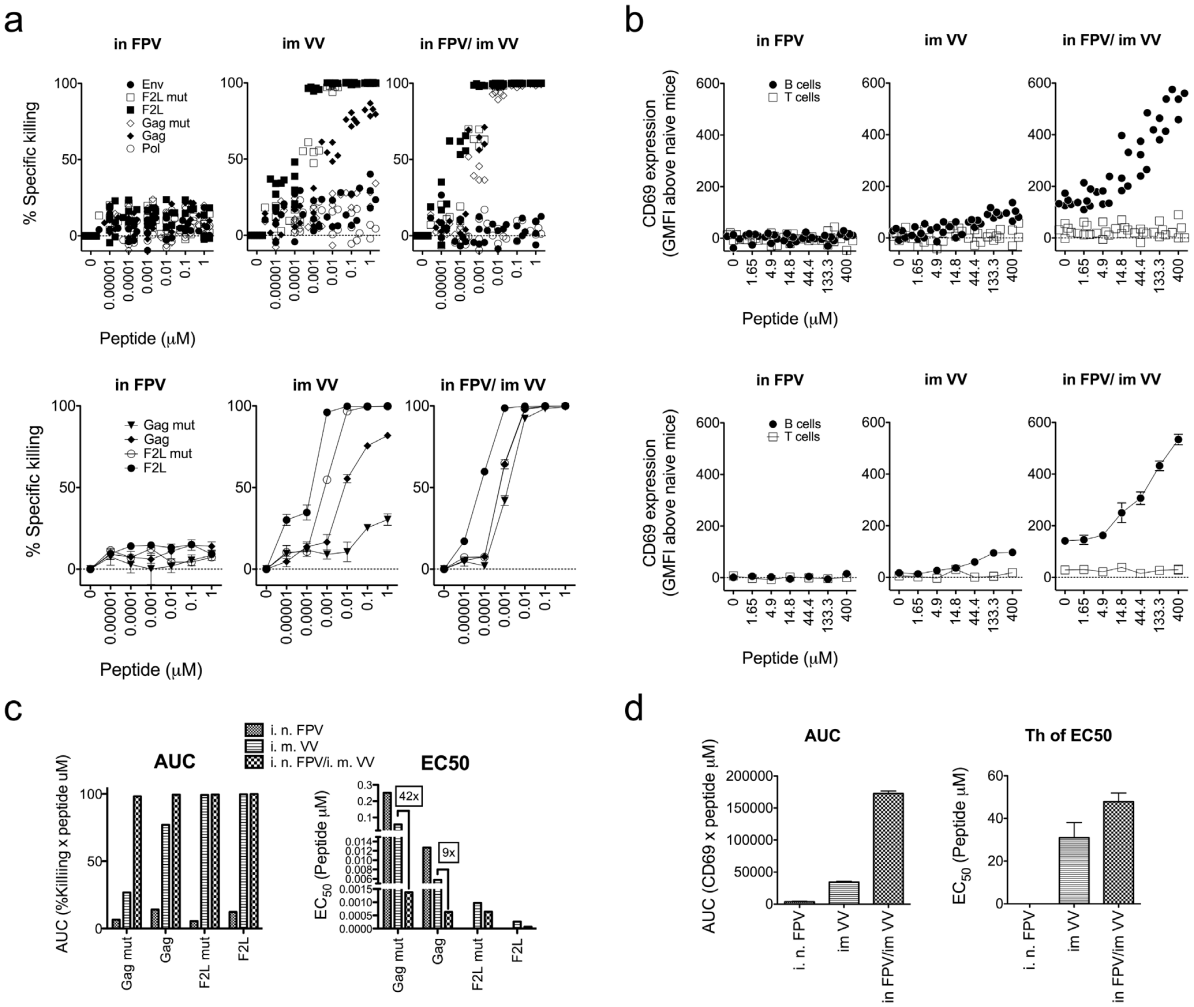
i.n.FPV-HIV/i.m.VV-HIV prime-boost vaccination improves the magnitude, functional avidity and epitope variant cross-reactivity of T-cell responses compared to prime or boost vaccinations alone. Mice were vaccinated i.n. with FPV-HIV and/or i.m. VV-HIV. Mice were vaccinated with 5×10^6^ PFU of each pox virus vaccine. Booster vaccinations were given 2 weeks post the previous vaccination. T-cell responses were assessed using 252-parameter FTAs as in Figure 3. a) % specific killing of FTA cells *in vivo* by CTL and b) T_H_ cell activity induced by prime, boost, and prime-boost vaccination regimes, showing all 6 intra-animal replicate responses (upper panels) and means and standard error of means (lower panels) to the various CTL epitopes. b) Mean and standard error of means from a). AUC and EC_50_ values of: c) CTL responses and: d) T_H_ cell responses. Values are only shown where EC_50_ values were calculable as described in the Methods. AUC and EC_50_ values are depicted as means from 5 intra-animal replicates. The results are representative of seven independent experiments.

Once vaccine strategies of interest have been identified from these heat map summary statistics, detailed analysis can be performed on particular vaccine regimes of interest. For example, from previous studies [13]–[16] the i.n. FPV-HIV/i.m. VV-HIV prime-boost vaccination strategy seemed particularly promising in generating HIV-specific CD8^+^ T-cells responses; and this was also found to be an outstanding regime in his study being in the FPV-HIV/VV-HIV prime-boost cluster. Therefore, detailed analysis of this prime-boost regime relative to the prime and boost vaccine components alone was performed. Although FPV-HIV alone vaccination induced negligible CTL responses against the 3 Gag epitopes (**Fig. 4**), the recombinant VV-HIV alone typically generated a good killing response to Gag, a low killing response to Gag mut and a low T_H_ response to Gag Th (**Fig. 4**). Despite generating negligible responses to the Gag epitopes, FPV vaccination dramatically improved Gag specific killing responses when given as a prime vaccination with a VV boost vaccination. The killing response to Gag pulsed targets increased to levels on a par with targets pulsed with F2L mut epitopes (**Fig. 4a and 4c**). Furthermore, there was a 9-fold functional avidity improvement against the Gag epitope in the prime-boost animals compared to vaccination with VV alone (**Fig. 4c**). What was also striking was the dramatic increase in response to the Gag mut epitope in the animals given the prime-boost regime both in terms of magnitude and functional avidity, with a 3-fold increase in cumulative killing and a 42-fold functional avidity improvement compared to responses generated to VV alone (**Fig. 4c**). There was also a 4-fold increase in cumulative T helper response towards the Gag Th epitope in these animals; although there was no detectable functional avidity change in the Gag Th response (**Fig. 4d**). This detailed analysis revealed that despite not generating a detectable immune response when given alone, FPV-HIV vaccination could dramatically influence the magnitude, functional avidity and cross-reactivity when used in a FPV-HIV/VV-HIV modality.

### Refinement of *in vivo* T-cell assays using FTA technology dramatically reduces the numbers of animal required for screening vaccine-induced T-cell responses

By using the 252-parameter FTA assay described here, the number of animals required to assess T-cell responses *in vivo* were dramatically reduced. This is highlighted in the vaccine screening experiment above. If 25 vaccine strategies (including a naïve control and 24 vaccinations) were assessed using a conventional 2-parameter *in vivo* CTL assay, against 7 distinct epitopes at 6 epitope concentrations and 6 replicates, the total number of host mice required would equal 6300 (**Table 2**). To perform this study using this number of animals, a limit of 50 animals per experiment might be achievable considering the number of events required during flow cytometry; and therefore a projected number of 126 experiments would have to be performed. Each of these experiments would use one additional animal (minimum) as a donor for target cells. Therefore, a total number of 6426 animals might be needed to perform the entire vaccine screen study over 126 experiments using a 2-parameter assay. This contrasts with the 45 animals required to perform a similar study using a 252-parameter FTA assay (**Table 2**). It should be noted that the experiments using the two different methodologies are not exactly congruent, with the replicates in the 2-parameter assay being inter-animal and those in the 252-parameter assay being intra-animal; it is also unlikely that the experiment would be practical with a 2-parameter assay in that comparisons between vaccination strategies performed in different experiments would not be ideal. Nevertheless, the FTA assay is a significant reduction and refinement of existing techniques that allows for potentially dramatic reduction in animal numbers when using *in vivo* T-cell assays.

**Table 2:** Animal number requirements for screening assays.

	Host mice					Donor mice	Total mice
	25 Immunizations	7 Epitopes	6 Epitope concentrations	6 Replicates	Total		
2 parameter conventional assay	25×	7×	6×	6×	6300	126	6426
252 parameter FTA assay	25×	1×	1×	1×	25	20	45
Fold difference in animals required					252	6.3	143

The number of animals required for *in vivo* T cell assays to screen the 25 vaccine strategies (including a naïve control and 24 vaccinations) depicted in Figure 3 using a conventional 2-parameter *in vivo* assay or a 252-parameter FTA *in vivo* assay.

## Discussion

The FTA is a unique assay that allows the simultaneous measurement of CTL killing and T_H_ activity against numerous target cells pulsed with a broad concentration range of several different MHC-I/II binding peptides *in vivo*. The versatility of this assay overcomes many of the limitations associated with other techniques for measuring CTL killing and T cell mediated B cell activation. For example, previously developed *in vivo* CTL killing assays [8]–[10], [17] are only able to measure the magnitude of the killing response in a single animal without providing any detailed measurements on functional avidity and epitope variant cross-reactivity. *In vitro* assays that measure T cell activity/avidity, such as the ^51^Cr-release assays, multi-parameter intracellular cytokine staining assays and ELISpot peptide dilution assays, typically require *ex vivo* stimulation of host effector T cells which can result in changes in functional avidity at a population level, due to preferential outgrowth of high avidity effector T cells [18]. Thus, the FTA assay is an *in vivo* assay with an excellent capacity to measure multiple parameters of T cell responses in a single animal.

The versatility of the FTA assay allowed the comprehensive screening of T cell responses generated from 24 different vaccination regimes against 7 distinct viral epitopes, which included HIV epitopes (Gag, Gag Th, Gag mut, Pol and Env) as well VV epitopes (F2L and F2L mut). From this analysis, heterologous prime-boost vaccination regimes, particularly those with FPV-HIV administered as the priming and VV-HIV administered as the booster vaccination, were found to be the most effective strategy in generating high magnitude CTL responses of high functional avidity and high epitope variant cross-reactivity. These results further substantiate our previous studies showing that amongst numerous prime-boost vaccination regimes involving the use of recombinant DNA, FPV-HIV and VV-HIV, the FPV-HIV/VV-HIV vaccination regime was the most effective in generating both high magnitude and high avidity HIV Gag-specific CD8^+^ T cell responses [13], [15], [16]. The synergistic improvement in responses to the HIV Gag epitopes seen in these heterologous prime-boost strategies may reflect the establishment of enhanced competition of HIV Gag epitopes for generating T cell responses over that of the dominant vector epitopes [19]. Since the immune system is not exposed twice to the dominant vector epitopes in these vaccine strategies, the vector epitope-induced responses are less able to compete with the memory responses generated against the HIV Gag epitopes post booster vaccination. This may then result in selective enrichment of high avidity HIV Gag-specific T cells [20]. These finding once again demonstrate that vector combination plays a critical role in vaccine efficacy. Interestingly, the FTA assay did not distinguish any vaccination route dependency with the FPV-HIV/VV-HIV vaccination strategy with regards to functional avidity profiles of CTLs in the systemic (splenic) compartment. Mucosal vaccination regimes have previously been shown to induce CTLs of higher avidity, particularly at mucosal sites, with greater protective immunity compared to the purely systemic delivery of vaccines [15], [21], [22]. Thus, it will be of great interest to use the FTA assay to assess how the different vaccine administration routes can alter the quality of mucosal T cell immunity. One caveat of the FTA assay in its current form is that it relies on target cells that are pulsed with known CTL and T_H_ epitopes rather than target cells that naturally present these epitopes from infecting pathogens. It will, therefore, be important to relate T cell responses measured with the FTA assay to microbe challenge studies or devise ways in which to use infected targets in the assay. In addition, since a large number of targets can be generated by the FTA assay, there is potential to use the method in screening MHC-binding peptide libraries for epitopes that are recognized by responding T cells, which can then be used in subsequent vaccine screening assay.

Recent studies indicate epitope variant cross-reactive T cell responses are of importance when dealing with diseases like HIV-1. Broad epitope variant cross-reactivity of CTLs has been observed in HIV patients who are ‘elite controllers’ [7]. Similar findings have been reported in hepatitis C virus patients, where clearance of the virus was associated with increased CTL epitope variant cross-reactivity and heightened functional avidity [18]. Interestingly, the FPV-HIV/VV-HIV vaccination regime that induced high avidity CTL also was able to generate strong CTL responses to the mutant Gag epitope, suggesting that high avidity CTL clones have greater capacity to recognize Gag escape mutant variants. In the context of an effective HIV-vaccine, recognition of Gag escape variants are of high significance [23]. Collectively, the current study demonstrates that FPV-HIV/VV-HIV heterologous prime-boost vaccination regimes have the hallmarks of an efficacious vaccination strategy that could induce good protective efficacy.

In conclusion, by using the FTA assay we have extensively evaluated a combination of vectors expressing HIV epitopes for their ability to generate high quality T cell responses *in vivo*. We have found that the magnitude, functional avidity and epitope variant cross-reactivity of CTLs is dependent on the vaccine vector combination and out of the 24 pox virus vaccine combinations tested, FPV-HIV/VV-HIV vaccination strategies induced the best immune outcomes. Overall, our data suggest that the FTA assay is an extremely valuable tool that can be used for large scale screening of vaccine combinations for pre-clinical testing in a more ethical and cost effective manner.

## Material and Methods

### Mice and Ethics statement

Male 6-10 week old BALB/c mice were purchased from the Australian Phenomics Facility, Australian National University (ANU). All animals were maintained and experiments were performed in accordance with the Australian NHMRC guidelines within the Australian Code of Practice for the Care and Use of Animals for Scientific Purposes and in accordance with guidelines approved by the Australian National University Animal Experimentation and Ethics Committee (AEEC). This study was approved by the AEEC and listed under ANU ethics protocol number A2011/018. All animals were monitored daily, infected mice were scored for signs of illness and weight loss and when showing signs of undue stress were ethically sacrificed by cervical dislocation in accordance with the above AEEC approved protocols.

### Peptides

Peptides included the MHC-I-binding peptides, SPYAAGYDL (F2L, an L^d^-restricted vaccinia virus (VV) epitope), SPGAAGYDL (F2L mut, a modified vaccinia Ankara virus epitope homologous to F2L), AMQMLKETI (HIV Gag, an immunodominant K^d^-restricted HIV Gag epitope [24]), AMQMLKDTI (HIV Gag mut, a HIV Gag subtype C variant of HIV Gag [25]), VGPTPVNII (HIV Pol, a D^d^-restricted HIV pol epitope [26]), RGPGRAFVTI (HIV envelop (Env), a D^d^-restricted HIV env epitope [27], and AMQMLEKTI (HIV neg, a modified HIV gag epitope that serves as a negative control [28]) and the MHC-II-binding peptide PVGEIYKRWIILGLN (Gag T_H_, a H-2^d^-restricted HIV Gag epitope [24]). Peptides were synthesized at the Biomolecular Resource Facility, John Curtin School of Medical Research, ANU.

### Vaccines

The recombinant FPV encoding HIV AE clade Gag, Pol and Env (FPV-HIV - 117a); recombinant VV encoding HIV AE clade Gag and Pol (VV-HIV - 336); recombinant VV encoding HIV AE clade Env (VV-HIV - 337); and wild-type VV Western Reserve were prepared as described previously [14], [29].

### Immunization and FTA target cell injection

Host mice were immunized with 5×10^6^ PFU of FPV-HIV - 117a, a combination of 2.5×10^6^ PFU of VV-HIV - 336 and 2.5×10^6^ VV-HIV - 337 or 5×10^6^ PFU wild-type VV Western Reserve. All pox-viral vector immunizations were performed via the intranasal (i.n.) or intramuscular (i.m.) routes as described in **Table 1** under mild isoflurane anaesthesia. Immediately prior to delivery the viruses were diluted in phosphate buffered saline (PBS) and sonicated 20–30 s to obtain an homogeneous viral suspension, intranasal FPV was given in a final volume of 20–25 µl and i.m. VV were delivered, 50 µl per quadriceps. [14]. The FTAs were injected i.v. into host mice following 6 days post immunization at a total cell number of 5×10^7^ and left *in vivo* for 18 hr before FTA cells were assayed for target cell help and killing.

### FTA preparation

CFSE, CTV and CPD dye labeling and construction of FTAs was as described previously [11], [30]. Briefly, 2 ml aliquots of equal numbers of splenocytes in 20 °C RPMI-1640 media (Invitrogen) supplemented with 10% fetal calf serum (FCS), were labeled with 0–65595 nM of CTV for 5 min. Cells were then split equally in up to 7 aliquots and labeled with 0–47269 nM of CFSE in a final volume of 1 ml of RPMI-1640 supplemented with 10% FCS for 5 min. After washing the cells once, they were then pulsed with 0–400 µM MHC-I or MHC-II-binding peptide epitopes for 1 hr at 37°C. To remove excess peptide, cells were sedimented through a 3.5 ml FCS cushion at 4°C, then washed with 10 ml of RPMI-1640 supplemented with 10% FCS at 4°C and cells then pooled together. To generate intra-animal replicates the pooled cell population was split into six 2 ml aliquots and labeled with 0–38500 nM of CPD, washed twice and all aliquots pooled. Cells were washed twice with 10 ml of RPMI 1640 supplemented with 5% FCS, pooled and washed once more. To allow the FTA to be detected in syngeneic BALB/c host mice a 4^th^ dye, DiI, was used to label all targets cells for their detection *in vivo*. DiI (Invitrogen) was used at 14 µM to label cells as described by the manufacturer. The FTA was then counted and resuspended at up to 25×10^7^ cells/mL ready for injection.

### Antibody labeling of cells

Splenocytes from mice were labeled with antibodies to delineate cell populations and included anti-CD4-PE-Cy7, anti-CD4-Brilliant Violet 605, anti-CD4-Alexa Fluor 700, anti-CD8α-APC-eFluor 780, anti-B220-PerCP-Cy5.5, anti-B220-Alexa Fluor 700, anti-CD69-PerCp-Cy5.5, and anti-CD69-Brilliant Violet 605, purchased from either BD Bioscience, eBioscience or Biolegend. Cell viability was assessed with the dye Hoechst 33258 (1 µg/ml, Calbiochem-Behring Corp.). Cells were labeled with antibodies and Hoechst 33258 for 20–30 min on ice and washed twice as previously described [31].

### Flow cytometry of cells

For flow cytometry analysis, cells were first filtered through a 70 µM mesh to remove cell clumps. Flow cytometry was performed using a Fortessa flow cytometer (Becton Dickinson). Post acquisition gating was used to analyze cell subsets using FlowJo software (Tree Star, OR). The flow cytometer underwent periodic quality control assessment using eight channel fluorescent beads throughout the period of experimentation.

### % Specific killing and T cell help calculations

The % specific killing and T cell help was assessed as previously described [11], [12], [28]. Briefly, for the CTL responses, the number of cells in each FTA cell cluster was calculated using FlowJo software and the % specific killing values were then generated using the following formula:
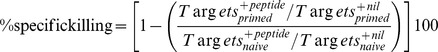



T cell help was assessed on the basis of CD69 upregulation on FTA B cell by antibody labeling and flow cytometry. Expression of CD69 in the Figures was calculated by subtracting the activation marker geometric mean fluorescence intensity (GMFI) on FTA B cells from naïve mice from that on FTA B cells from the vaccinated mice.

### Statistical analysis

Area under the curve (AUC), EC_50_ (peptide concentrations giving half maximal response) and the Mann-Whitney nonparametric two-tailed test was performed using GraphPad Prism Software 5.0f (Graphpad Inc).

## Supporting Information

Figure S1 **Raw data from screening of HIV-1 pox virus vaccination regimes for high magnitude, high-functional avidity and high epitope variant cross-reactive T cell responses** ***in vivo*** **.** Mice were vaccinated with 24 different vaccination regimes as in Figure 3. T cell responses were assessed using a 252-parameter FTAs comprised of fluorescent target cells pulsed with 6 different concentrations of the MHC-I binding peptides F2L, F2L mut, HIV Gag, HIV Gag mut, HIV Pol and HIV Env, and the MHC-II binding peptide Gag Th and this repeated 6 times to generate 6 intra-animal replicates. % specific killing data from all intra-animal replicates (a) and associated means and standard error of means (b). T_H_ cell activity data from all intra-animal replicates (c) and associated means and standard error of means (d). e) Cumulative magnitude of T cell responses as AUC plotted as a heat map depicting the mean CTL (upper panels) or mean T_H_ cell (lower panel) responses from 6 intra-animal replicates at each concentration of epitope used to pulse target cells (in µM).
(DOC)
