# Supplementary figures and images for: Use of an In Vivo FTA Assay to Assess the Magnitude, Functional Avidity and Epitope Variant Cross-Reactivity of T Cell Responses Following HIV-1 Recombinant Poxvirus Vaccination
Source: PLoS One. 2014 Aug 29;9(8):e105366. doi: 10.1371/journal.pone.0105366 (PMC4149432; doi:10.1371/journal.pone.0105366)

**Supporting Information Figure S1**


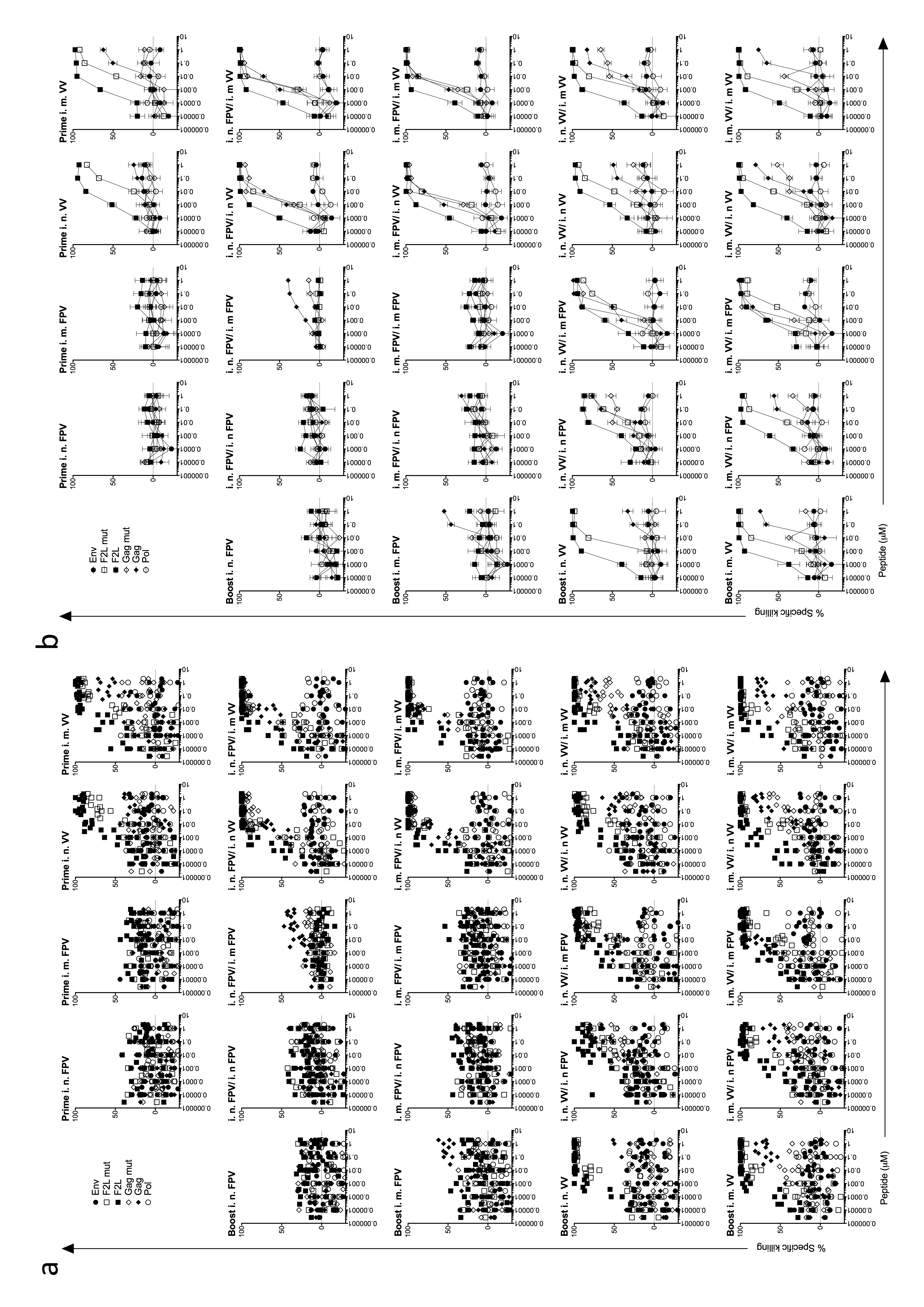


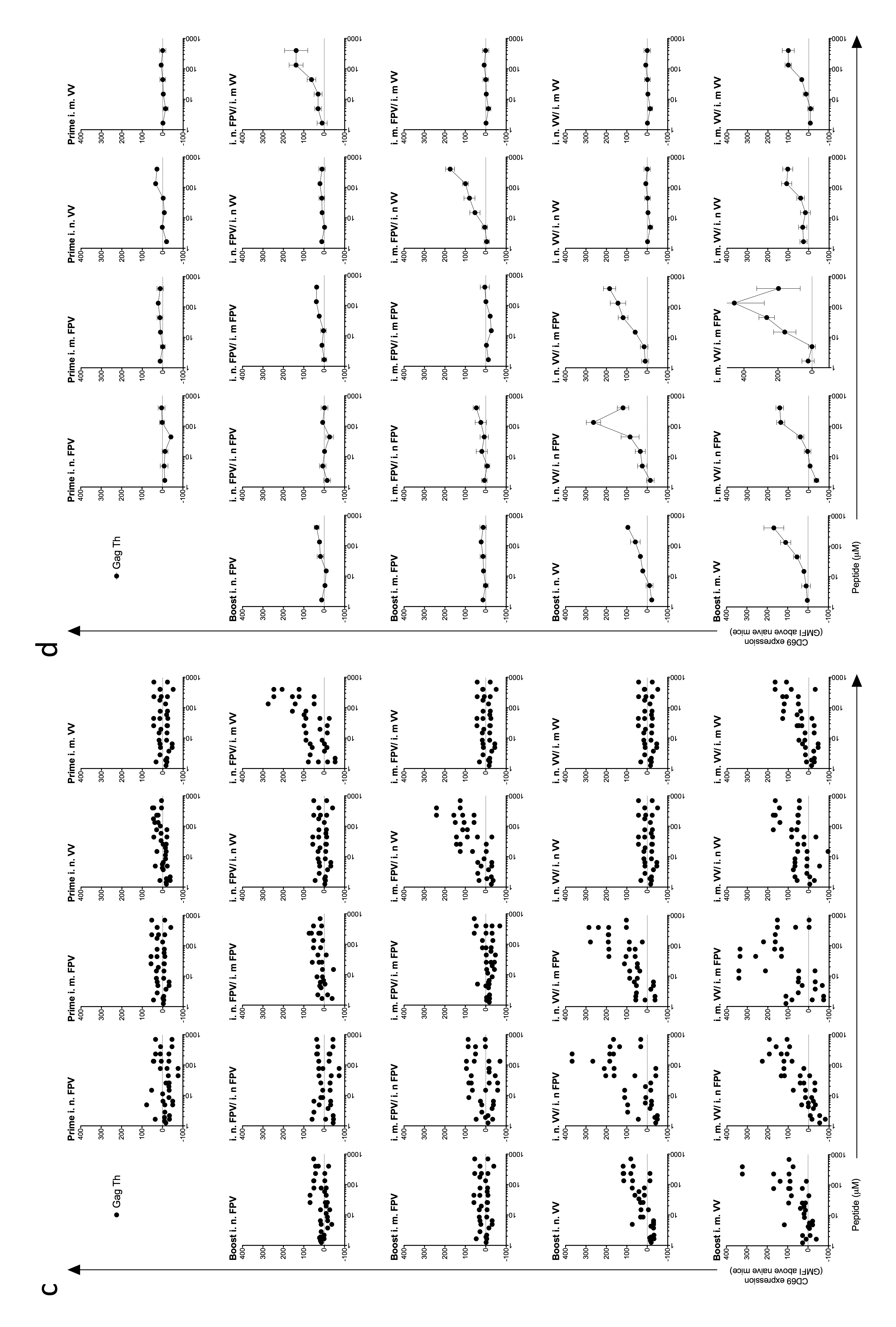


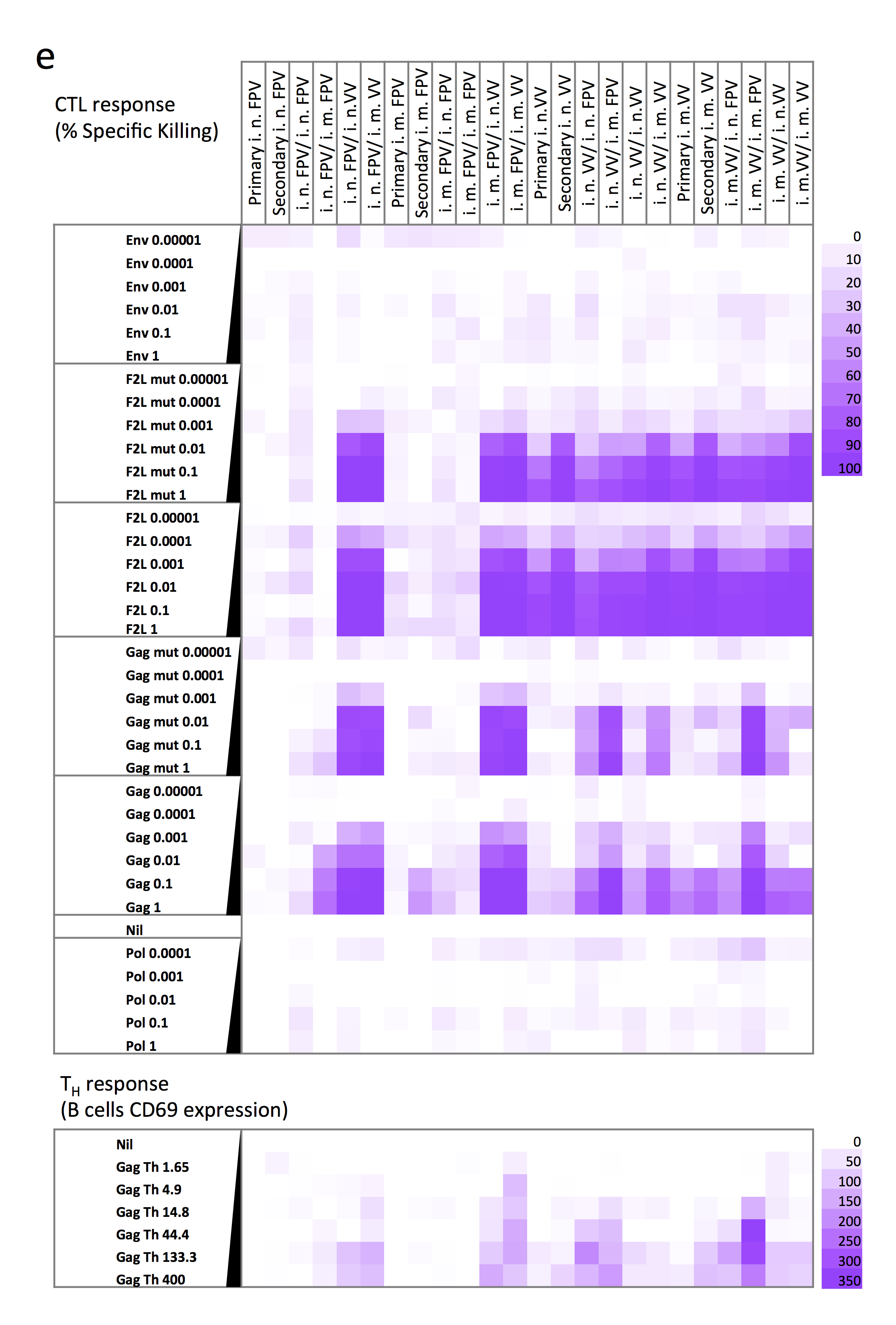

Supplement: Figure S1 — Raw data from screening of HIV-1 pox virus vaccination regimes for high magnitude, high-functional avidity and high epitope variant cross-reactive T cell responses in vivo . Mice were vaccinated with 24 different vaccination regimes as in Figure 3. T cell responses were assessed using a 252-parameter FTAs comprised of fluorescent target cells pulsed with 6 different concentrations of the MHC-I binding peptides F2L, F2L mut, HIV Gag, HIV Gag mut, HIV Pol and HIV Env, and the MHC-II binding peptide Gag Th and this repeated 6 times to generate 6 intra-animal replicates. % specific killing data from all intra-animal replicates (a) and associated means and standard error of means (b). TH cell activity data from all intra-animal replicates (c) and associated means and standard error of means (d). e) Cumulative magnitude of T cell responses as AUC plotted as a heat map depicting the mean CTL (upper panels) or mean TH cell (lower panel) responses from 6 intra-animal replicates at each concentration of epitope used to pulse target cells (in µM). (DOC) [file pone.0105366.s001.doc]
